# Conventional and transepithelial corneal cross-linking for patients with keratoconus

**DOI:** 10.1371/journal.pone.0195105

**Published:** 2018-04-05

**Authors:** Xiaoyu Zhang, Jing Zhao, Meiyan Li, Mi Tian, Yang Shen, Xingtao Zhou

**Affiliations:** Eye and ENT Hospital of Fudan University, Myopia Key Laboratory of the Health Ministry, Shanghai, China; Save Sight Institute, AUSTRALIA

## Abstract

Previous studies investigating the effectiveness of conventional corneal collagen cross-linking (CXL) and transepithelial CXL in keratoconus treatment have reported conflicting outcomes. Therefore, we conducted a meta-analysis to compare the effectiveness of these treatments. We searched MEDLINE, EMBASE, and Cochrane Central Register of Controlled Trials for prospective randomized controlled trials (RCTs) with no restrictions. We included visual acuity (corrected distance visual acuity, uncorrected distance visual acuity) and corneal keratometry (K) as primary outcome parameters, and spherical equivalent, central corneal thickness (CCT), and endothelial cell density, as secondary parameters. We finally included seven reports (including six RCTs involving 305 participants and 344 eyes). Our analysis revealed significant postoperative differences in average K and CCT values between conventional and transepithelial CXL-treated patients [K: weighted mean difference (WMD) = 0.79, 95% confidence interval (CI) = 0.04–1.53, *p* = 0.04; CCT: WMD = 4.53, 95% CI = 0.42–8.64, *p* = 0.03]. In contrast, we did not find any significant differences in visual acuity, flattest K value, steepest K value, cylinder K value, apex K value, spherical equivalent, or endothelial cell density between groups. In conclusion, transepithelial CXL has a more protective influence on corneal thickness than conventional CXL, and results in lesser postoperative corneal flattening. Further investigation of the clinical outcomes of transepithelial CXL is required.

## Introduction

Keratoconus is a severe corneal disorder involving progressive corneal thinning, ectasia, and induced irregular astigmatism, which can lead to impaired vision [1, 2]. Available treatment options include rigid contact lenses, intracorneal ring segments, and keratoplasty. For advanced keratoconus cases, corneal cross-linking (CXL) is now widely used due to the resulting enhancements in mechanical strength, provision of biochemical stability, and slowing or prevention of progression.

The conventional “epithelium-off” protocol has been applied in the majority of previous clinical studies [3–6]. Transepithelial CXL, also known as “epithelium-on” CXL, is now widely employed due to its comfort and safety, as it avoids epithelial removal, thus reducing risk of complications. Ever since their widespread use, there have been extensive studies on the efficacy of both treatments; however, the clinical efficiency of transepithelial CXL remains controversial [7, 8]. The absence of clinical trials and disagreement among prospective randomized controlled trials (RCTs) have led to a lack of definitive evidence on the treatment efficacy of these procedures for patients with keratoconus. Therefore, we performed a meta-analysis of prospective RCTs to compare the efficacy of conventional and transepithelial CXL on keratoconus treatment.

## Methods

We performed a meta-analysis of prospective RCTs in accordance with the PRISMA statement in the existing literature.

### Search strategy

Two reviewers searched independently through MEDLINE, EMBASE, and Cochrane Central Register of Controlled Trials (up to July 1, 2017) for prospective RCTs, with no restrictions, and evaluated studies for their content. For each database, we combined search terms by using Medical Subject Heading terms and free key words, including “cross linking,” “crosslinking,” “cross-linkage,” “cross-linking,” “cross-linking reagents,” and “keratoconus” The reference lists of all identified articles were also hand-searched. Beyond that, the included articles were scrutinized for other potentially relevant studies.

### Selection criteria

The inclusion criteria comprised the following: 1) RCTs; 2) patients diagnosed with keratoconus (progression of keratoconus) with a mean age > 18 years; 3) comparison of the conventional (standard Dresden protocol, UVA exposure: 5.4 J/cm^2^ for 9 to 30 minutes) and transepithelial processes (preserving epithelium, including iontophoresis-assisted technique); and 4) reported changes in distance visual acuity, refractive parameters, corneal convexity parameters, central corneal thickness (CCT), endothelial cell density (ECD), and intraocular pressure (IOP). We resolved any discrepancies by arbitration and negotiated agreement on study inclusion. Articles on CXL combined with other treatments, such as photorefractive keratectomy, deep anterior lamellar keratectomy procedure, or intrastromal corneal ring segments, were excluded during the initial review phase. Reviews, abstracts, case-series, observational studies, and animal studies were excluded. Studies including patients with secondary keratoconus or ones treated with the Lasik procedure were also excluded.

### Data extraction

Two investigators separately extracted all essential data and resolved any discrepancies by arbitration. The following data were abstracted from the included studies: the last name of the first author, country, publication year, size of study population, follow-up time, participants’ age, number of treated eyes, and UVA radiation wavelength and treatment.

### Outcome measures and quality assessment

Primary main outcome parameters in our study included pre- and postoperative corrected distance visual acuity (CDVA; logMAR), uncorrected distance visual acuity (UDVA; logMAR), corneal convexity parameters (steepest simulated keratometry [K] value [K-steepest], flattest simulated keratometry value [K-flattest], average simulated keratometry value [K-avg], cylinder simulated keratometry value [K-cyl], and apex simulated keratometry value. Secondary results included spherical equivalent (SE) and CCT. Finally, ECD and IOP were set as the last observation indicator to evaluate the safety of surgery. Postoperative complications for two CXL procedures were also analyzed.

We determined the efficacy of the two procedures by the changes in clinical outcomes from baseline to endpoint. We recorded change-value, which was presented in the included studies. As for studies separately presenting pre- and postoperative outcome values, we processed the extracted data and calculated the changes in different outcome values in order to perform the analysis. The quality of included trials was assessed by the Jadad score [9]. We resolved data interpretation discrepancies through discussion with a third reviewer and evaluation of the original articles.

### Data synthesis and statistical analysis

A meta-analysis was performed for the included data. For continuous measurements, the weighted mean difference (WMD) and corresponding 95% confidence intervals (CIs) were used. For the included RCTs, the WMD was calculated by the difference in the mean change in the conventional and the transepithelial CXL-treated groups. The results were evaluated as mean ± standard deviation. Heterogeneity between studies was assessed using *I*^2^ statistics [10]. According to Higgins *et al*., *I*^2^ values of 25%, 50%, and 75% were classified as low, moderate, and high heterogeneity, respectively [11]. For the meta-analysis, the random-effects model was used for all the analyses [12]. Begg and Egger’s regression test was used for the detection of potential publication bias. If this test showed a publication bias, we further performed the “trim and fill” analysis [13, 14], by which we assessed the potential hypothetical “missing” studies and related WMDs. We then re-calculated the WMDs, by including the assigned WMDs of hypothetical missing studies. Begg and Egger’s regression test and “trim and fill” analysis were performed using Stata (version 12.0, StataCorp, College Station, TX, USA), while other analyses were conducted by using the RevMan software (version 5.3; Cochrane Collaboration, Oxford, UK). Probability values < 0.05 were considered statistically significant.

## Results

**Fig 1:**
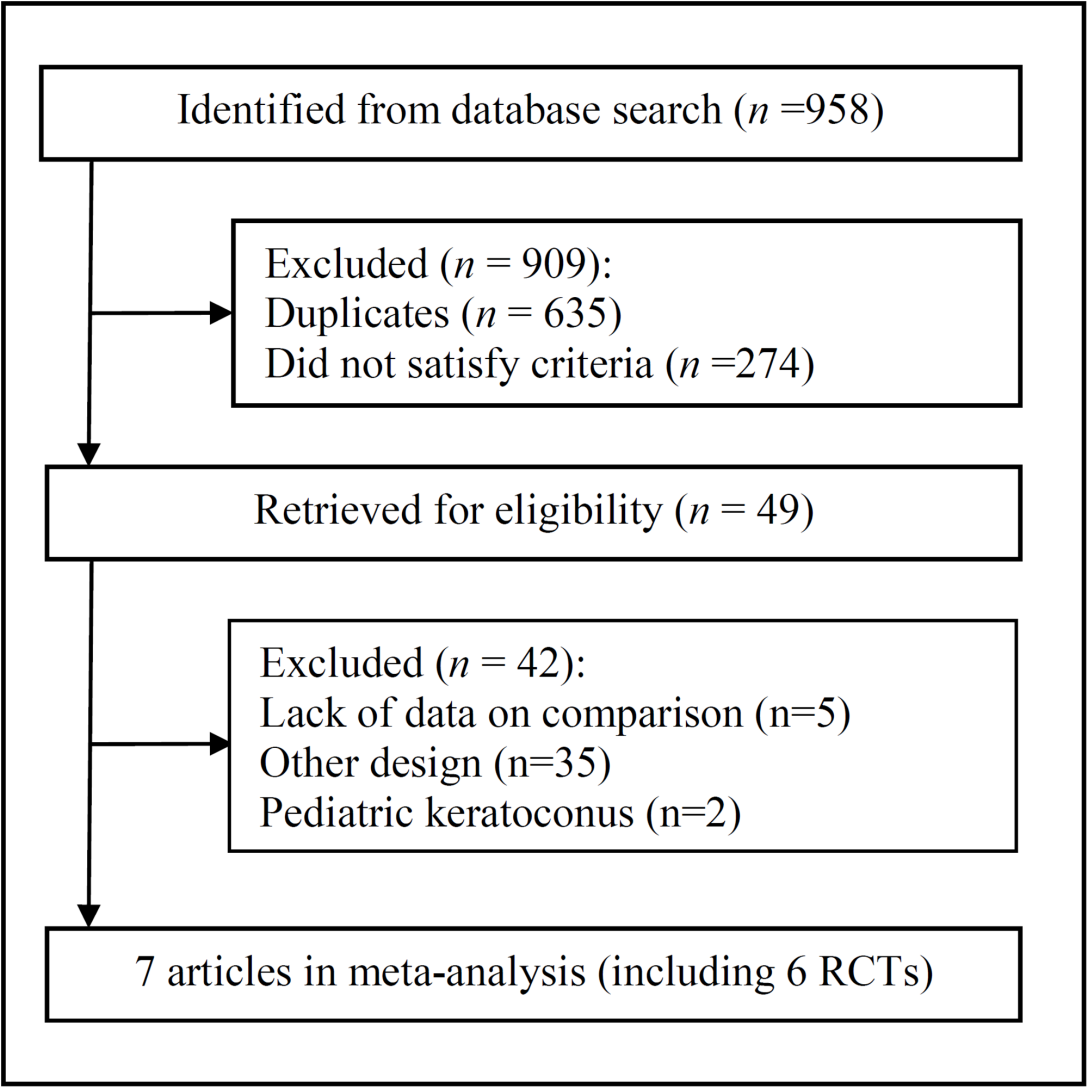
Flowchart of studies included in the meta-analysis.

The selection process of included studies is shown in Fig 1. We identified 958 articles from the initial database search. After evaluating the abstract of each study, we excluded 635 duplicated studies and 274 studies not satisfying the criteria. This resulted in a remainder of 49 articles. Subsequently, a few studies were excluded due to lack of data on comparison (n = 5), other design (n = 33), or involving CXL treatment in pediatric patients with keratoconus (n = 2). Two articles were published in 2016 and 2017 based on a study conducted at the clinical trials center of the Istituto di Ricovero e Cura a Carattere Scientifico Fondazione G.B. Bietti (Rome, Italy). After careful evaluation, the article with the longer follow-up was included in our study [15], and SE was conducted based on the article published in 2016, since the one published in 2017 did not include this data [16]. Finally, six RCTs (7 articles) were included in the meta-analysis [15–21].

### Characteristics of included studies

The main characteristics of the included RCTs are described in Table 1. There were 183 eyes included in the transepithelial CXL group and 161 eyes included in the conventional CXL group. Studies were conducted in Italy, the Netherlands, India, and Russia and were reported between 2013 and 2017. The duration of follow-ups ranged from 6 to 24 months, the mean participant age ranged from 22.35 to 31.05 years, and the Jadad scores of the included RCTs ranged from 2 to 4.

**Table 1:** Characteristics of 6 randomized controlled trials included in analysis. (K, keratometry; D, diopter; SE, spherical equivalent; epi-off, conventional CXL; epi-on, transepithelial CXL).

Study (Year)	Location (Follow-up)	CXL Protocol	Inclusion criteria of progressive keratoconus	Postoperative Treatment	Postoperative Complications	Age (epi-off/epi-on)	No. eyes (epi-off/epi-on)	Male (epi-off/epi-on)	UVA irradiance (mW/cm^2^)/ UVA wavelength (nm)	Jadad score
**Mastropasqua et al**. **(2013)**	Italy (12 months)	Epi-off CXL: riboflavin for 15mins, UVA irradiation for 30mins Epi-on CXL: riboflavin for 30mins, UVA irradiation for 30mins	Mean central K-change of ≥1.5D in 3 topographies in 6 months	NA	NA	23/23	20/20	NA/NA	3/370	2
**Nawaz et al**. **(2015)**	India (6 months)	Epi-off CXL: riboflavin for 30mins, UVA irradiation for 30mins Epi-on CXL: riboflavin for 30mins, UVA irradiation for 30mins	>1D steep K-change in 12 months or >0.5D in 6 months	Topical moxifloxacin, predacetate drops. Oral nonsteroidal anti-inflammatory drugs and soft bandage contact lens applied in epi-off CXL group.	NA	23.95/22.35	20/20	15/17	3/765	3
**Rossi et al**. **(2015)**	Italy (12 months)	Epi-off CXL: riboflavin for 30mins, UVA irradiation for 30mins Epi-on CXL: riboflavin and UVA irradiation for 30mins	Worsen topographic, pachymetric, or aberrometric in 6 months	Topical tobramycin, dexamethasone phosphate, lubricating drops and therapeutic contact lens in epi-off group, no contact lens and corticosteroid drops were instilled in epi-on group.	No ocular or systemic adverse events were observed.	30.4/28	10/10	5/6	3/370	2
**Soeters et al**. **(2015)**	Netherlands (12 months)	Epi-off CXL: riboflavin for 30mins, UVA irradiation for 30mins Epi-on CXL: riboflavin for 30mins, UVA irradiation for 30mins	Max/steep/mean K-change and/or topographic cylinder-change ≥0.5D in 6–12 months	Antibiotic drops, preservative-free artificial tears, nonsteroidal anti-inflammatory drops (first week), and topical steroids (from second week) were applied. Oral pain medication and bandage lensapplied in epi-off group.	Herpes simplex keratitis, sterile infiltrate, epithelial healing problems in epi-off group. No adverse events in epi-on group. 23% detected progression in epi-of group, and 14% retreated by epi-off CXL.	24/24	26/35	19/28	3/NA	4
**Bikbova et al**. **(2016)**	Russia (24 months)	Epi-off CXL: riboflavin for 30mins, UVA irradiation for 30mins Epi-on CXL: riboflavin for 10mins by iontophoresis, UVA irradiation for 30mins	Steep K-change >1D in manifest cylinder, or >0.5D in manifest SE	Antibiotics and topical steroids for epi-off group. Corticosteroid for epi-on group.	Slight stromal edema and epithelial healing problem in epi-off group. No adverse events in epi-on group. One patient detected progression in epi-on group.	30/28	73/76	NA/NA	3/370	3
**Lombardo et al**. **(2016)**	Italy (6 months)	Epi-off CXL: riboflavin for 30mins, UVA irradiation for 30mins Epi-on CXL: riboflavin for 5mins by iontophoresis, UVA irradiation for 9mins	Max K-change of ≥1D in 12 months	Ofloxacin, sodium hyaluronate, and fluorometholone acetate for both groups. Bandage lens applied in epi-off group.	Tearing and photophobia was reported in epi-off group.	29.4/31.05	12/22	8/18	3/370 for epi-off CXL 10/370 for epi-on CXL	4
**Lombardo et al**. **(2017)**	Italy (12 months)	Epi-off CXL: riboflavin for 30mins, UVA irradiation for 30mins Epi-on CXL: riboflavin for 5mins by iontophoresis, UVA irradiation for 9mins	Max K-change of ≥1D in 12 months	Ofloxacin, sodium hyaluronate, and fluorometholone acetate for both groups. Bandage lens applied in epi-off group.	Cornea scars was reported in epi-off group.	29.4/31.0	12/22	NA	3/370 for epi-off CXL 10/370 for epi-on CXL	4

The conventional CXL procedure in all included studies followed the Dresden protocol (central 9.0-mm-diameter corneal epithelium was removed, riboflavin 0.1% drops were instilled for 30 minutes, and eyes were irradiated with UVA for 30 minutes at an irradiance of 3 mW/cm^2^). The transepithelial CXL procedure varied; irradiation ranged from 3 to 10 mW/cm^2^, while iontophoresis was used in two studies to help riboflavin saturation (Bikbova and Lombardo) [15–17]. Regarding postoperative treatment, topical anti-biotic drops, nonsteroidal anti-inflammatory or steroid drops, and artificial tears were generally applied to reduce postoperative reactions, then tapered to zero. A bandage lens was placed in the conventionally-treated group in Soeters and Lombardo’s study [15, 16, 21]. (Table 1)

The inductive analysis of past studies helped us to divide the postoperative clinical efficacy indicators of our study into the following three categories: (1) main clinical indicators, including visual acuity (UDVA, CDVA) and corneal keratometry obtained from corneal topography; (2) secondary indicators, including SE and CCT; and (3) ECD, as an indicator of the safety of surgery. Postoperative complications for the two CXL procedures were also discussed.

### Main analysis

#### Visual acuity

**Fig 2:**
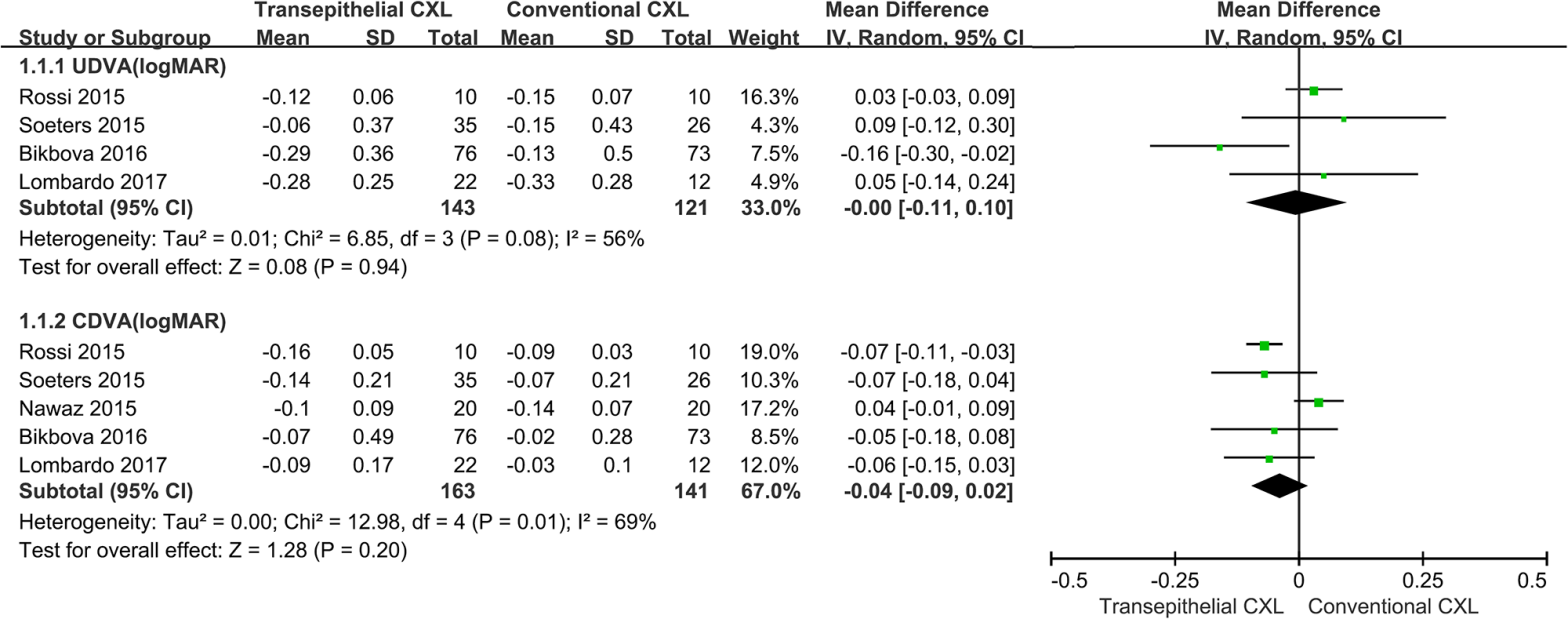
Changes in corrected distance visual acuity (CDVA [logMAR]) and uncorrected distance visual acuity (UDVA [logMAR]) between conventional and transepithelial corneal crosslinking-treated patients. WMD, weighted mean difference.

Four studies compared the UDVA(logMAR) between the conventional and transepithelial CXL-treated groups [15, 17, 20, 21]. Forest plot changes in UDVA outcomes between the two groups are provided in Fig 2. There were no significant differences between the two groups (WMD = 0, 95% CI = -0.11–0.10, *p* = 0.94), but a moderate heterogeneity was found among the studies (*p* = 0.08, *I*^*2*^ = 56%). Sensitivity analysis showed that the main source of heterogeneity was the study by Bikbova *et al* [17]. After removal of this study, no heterogeneity was observed in the remaining studies (*p* = 0.85, *I*^*2*^ = 0%). However, the results remained unchanged (WMD = 0.04; 95% CI = -0.02–0.09; *p* = 0.19). The Egger and Begg’s tests demonstrated a lack of publication bias (*p* = 0.095 for Egger’s test; *p* = 0.497 for Begg’s test).

Five studies compared the CDVA(logMAR) between the conventional and transepithelial CXL-treated groups [15, 17, 19–21]. Forest plot changes in CDVA outcomes between the two groups are presented in Fig 2. There were no significant differences in CDVA changes between groups (WMD = -0.04; 95% CI = -0.09–0.02; *p* = 0.20). A moderate heterogeneity was found among the studies (*p* = 0.01, *I*^*2*^ = 69%). The Egger and Begg’s tests demonstrated a lack of publication bias (*p* = 0.424 for Egger’s test; *p* = 0.327 for Begg’s test). Sensitivity analysis showed that the main source of heterogeneity was the study by Nawaz *et al* [19]. After this study was removed, no heterogeneity was observed in the remaining studies (*p* = 0.99, *I*^*2*^ = 0%); however, the results changed (WMD = -0.07; 95% CI = -0.10-(-0.04); *p* < 0.001).

#### Corneal keratometry

**Fig 3:**
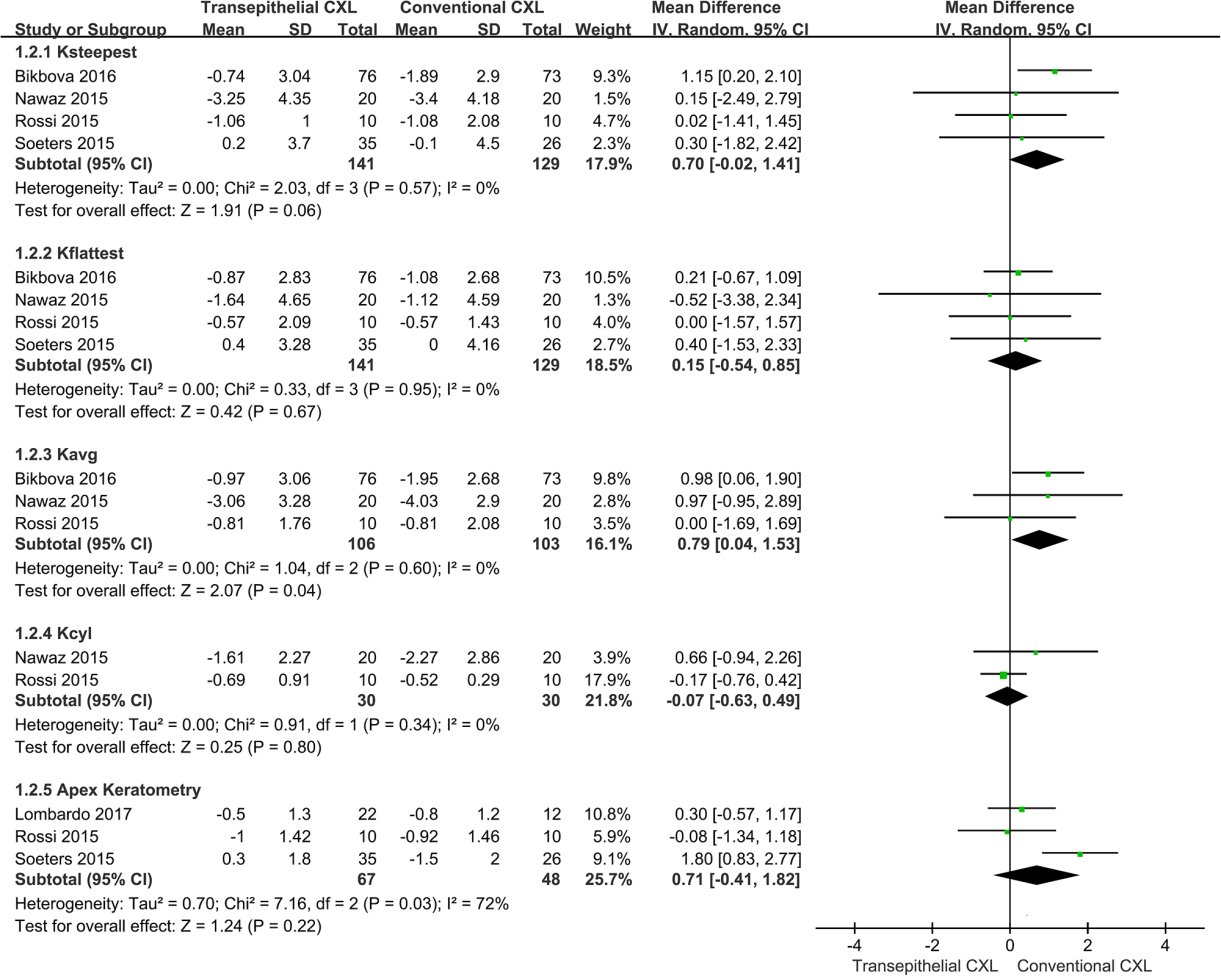
Changes in steepest keratometry (K) value (K-steepest), flattest K value (K-flattest), average K value (K-avg), cylinder K value (K-cyl), and apex K between conventional and transepithelial corneal crosslinking-treated patients. WMD, weighted mean difference.

Four studies compared the K-steepest between the conventional and transepithelial CXL-treated groups (Fig 3) [17, 19–21]. There was no significant difference in K-steepest changes between the two groups (WMD = 0.70; 95% CI = -0.02–1.41; *p* = 0.06) and no heterogeneity between studies (*p* = 0.57, *I*^*2*^ = 0%). The Egger and Begg’s tests demonstrated a lack of publication bias (*p* = 0.108 for Egger’s test; *p* = 1.000 for Begg’s test).

Four studies compared the K-flattest between the conventional and transepithelial CXL-treated groups (Fig 3) [17, 19–21]. There was no significant difference in K-flattest changes between the two groups (WMD = 0.15; 95% CI = -0.54–0.85; *p* = 0.67) and no heterogeneity between studies (*p* = 0.95, *I*^*2*^ = 0%). The Egger and Begg’s tests revealed a lack of publication bias (*p* = 0.436 for Egger’s test; *p* = 0.497 for Begg’s test).

Three studies compared the K-avg between the conventional and transepithelial CXL-treated groups (Fig 3) [17, 19, 20]. There was a significant difference in K-avg between the two groups (WMD = 0.79; 95% CI = 0.04–1.53; *p* = 0.04) with no heterogeneity between studies (*p* = 0.60, *I*^*2*^ = 0%). The Egger and Begg’s tests revealed no publication bias (*p* = 0.365 for Egger’s test; *p* = 0.117 for Begg’s test). The sensitivity analysis showed that after removal of the study by Bikbova *et al*. [17], the result changed (WMD = 0.42; 95% CI = -0.84–1.69; *p* = 0.51).

Two studies compared the K-cyl between the conventional and transepithelial CXL-treated groups (Fig 3) [19, 20]. There were no significant differences in K-cyl changes between the two groups (WMD = -0.07; 95% CI = -0.63–0.49; *p* = 0.80) with no heterogeneity between studies (*p* = 0.34, *I*^*2*^ = 0%).

Three studies compared apex K between the conventional and transepithelial CXL-treated groups (Fig 3) [15, 20, 21]. There were no significant differences between the two groups (WMD = 0.71; 95% CI = -0.41–1.82; *p* = 0.22) with high heterogeneity between studies (*p* = 0.03, *I*^*2*^ = 72%). Sensitivity analysis showed that after removal of the study by Soeters *et al*. [21], the heterogeneity disappeared (*p* = 0.63, *I*^*2*^ = 0%), and the results remained unchanged (WMD = -0.18; 95% CI = -0.54–0.89; *p* = 0.63). The Egger and Begg’s tests showed no publication bias (*p* = 0.141 for Egger’s test; *p* = 0.117 for Begg’s test).

### Secondary analysis

**Fig 4:**
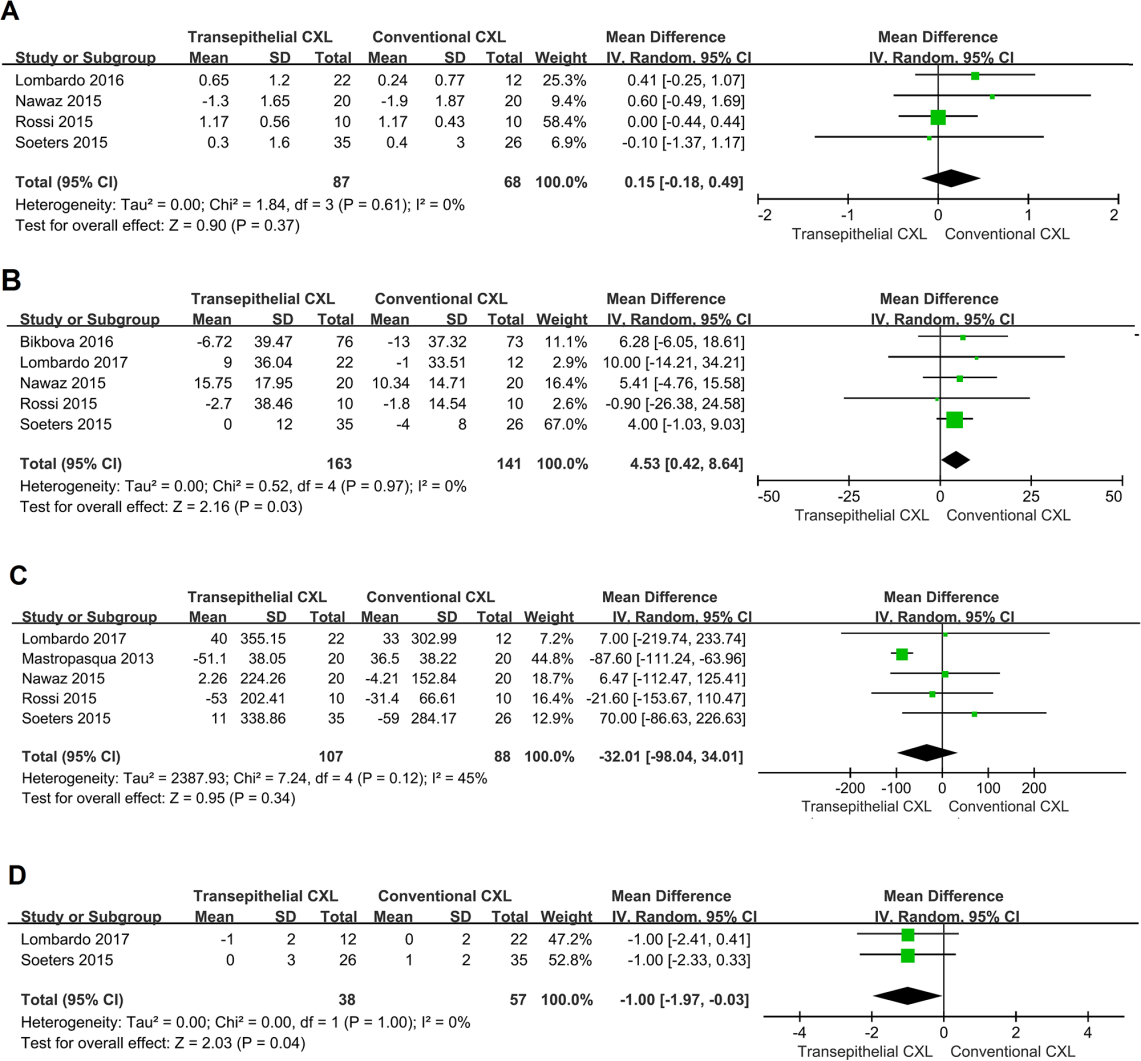
Secondary analysis. Changes in **A:** spherical equivalent (SE), **B:** central corneal thickness (CCT), **C:** endothelial cell density (ECD), and **D:** intraocular pressure (IOP) between conventional and transepithelial corneal cross-linking-treated patients. WMD, weighted mean difference.

Four studies compared the SE between the conventional and transepithelial CXL-treated groups (Fig 4A) [16, 19–21]. There were no significant differences in SE changes observed between the two groups (WMD = 0.15; 95% CI = -0.18–0.49; *p* = 0.37) with no heterogeneity between studies (*p* = 0.61, *I*^*2*^ = 0%). The Egger and Begg’s tests revealed no publication bias (*p* = 0.671 for Egger’s test; *p* = 0.497 for Begg’s test).

Five studies compared the CCT between the conventional CXL and transepithelial CXL groups (Fig 4B) [15, 17, 19–21]. There was a significant difference in CCT between the two groups (WMD = 4.53; 95% CI = 0.42–8.64; *p* = 0.03) with no heterogeneity between studies (*p* = 0.97, *I*^*2*^ = 0%). The Egger and Begg’s tests revealed no publication bias (*p* = 0.847 for Egger’s test; *p* = 0.327 for Begg test).

Five studies compared the ECD between the conventional and transepithelial CXL-treated groups (Fig 4C) [15, 18–21]. There were no significant differences in ECD changes between the two groups (WMD = -32.01; 95% CI = -98.04–34.01; *p* = 0.34) with moderate heterogeneity (*p* = 0.12, *I*^*2*^ = 45%). The Begg’s test revealed the existence of potential publication bias (*p* = 0.05); however, the Egger test revealed no publication bias (*p* = 0.296). The “trim and fill” analysis showed that there was one potentially missing study and the results remained unchanged. The sensitivity analysis demonstrated that, after removal of the study by Mastropasqua *et al*. [18], the result remained unchanged (WMD = 11.73; 95% CI = -61.16–84.62; *p* = 0.75) with no heterogeneity between studies (*p* = 0.85, *I*^*2*^ = 0%).

Two studies compared the IOP between the conventional CXL and transepithelial CXL groups (Fig 4D) [15, 21]. There were significant differences in IOP changes between the two groups (WMD = -1.00; 95% CI = -1.97-(-0.03); *p* = 0.04) with no heterogeneity between studies (*p* = 1.00, *I*^*2*^ = 0%).

### Characteristics of postoperative complications

Stromal edema was generally observed up to 7 days after the CXL treatment. In the conventional CXL-treated group, adverse events, including herpes simplex keratitis, sterile infiltrate, delayed epithelial healing problems, and long-term corneal haze, were observed (by Soeters *et al*.) [21]; however none of these resulted in any visual acuity loss. Postoperative symptoms, such as tearing, photophobia, and perioperative pain, were observed by Lombardo (using iontophoresis) [15, 16], Nawaz [22], and Bikbova *et al*. [17], but were generally improved during follow-up. Fewer postoperative symptoms were observed in the transepithelial CXL-treated group in these studies.

Regarding keratoconus progression, only one patient (1.3%) in Bikbova’s study [17]showed progression in the 24-month follow-up after transepithelial CXL. In another study, conducted by Soeters *et al*. [21], 23% of the cases treated with transepithelial CXL showed continued keratoconus progression, among which, one eye was re-treated by conventional CXL after 10 months, due to increased maximal K (4.7 diopter), while four eyes were re-treated after 1 year.

None of the included studies reported cases that were inevitably ended by keratoplasty during follow-up.

## Discussion

We included seven articles from six RCTs (305 participants, 344 eyes) in a meta-analysis to compare the effectiveness of conventional and transepithelial CXL treatment in patients with keratoconus. There were significant postoperative differences in the K-avg, CCT, and IOP values between the two groups. No significant differences were revealed from the pooled results for the UDVA, CDVA, K-steepest, K-flattest, K-cyl, apex-K, SE, or ECD.

Our meta-analysis indicates that transepithelial CXL is more protective than conventional CXL for corneal thickness, while it decreases postoperative corneal flatting, evident by the smaller reduction in the K-avg. We believe that transepithelial CXL is more suitable, less risky, and safer for patients with progressive keratoconus, who have a relatively thin cornea, since preserving the epithelium also protects the corneal endothelium, at least to some extent. On the other hand, conventional CXL might be more effective in reducing corneal K in patients with thicker and steeper corneas and advanced disease progression. We do not recommend that transepithelial CXL should completely replace the standard “epithelium off” CXL, as there are continued ectatic progression cases, however, we suggest that it is as a more comfortable, less-complicated procedure, and a beneficial choice for patients with thin corneas or unfit for standard “epithelium-off” CXL treatment.

As a noninvasive treatment for keratoconus, corneal CXL is now widely accepted. Since the first clinical report on CXL by Wollensak *et al*. in 2003 [3], several studies have provided evidence that this procedure halts progressive keratoconus, and it may have a flattening function on the cornea [3, 23–25]. Meanwhile, the prevalence of stromal scarring ranges from 2.8 to 3.4% in patients that undergo conventional CXL [26–28]. Other complications, such as bacterial keratitis and sterile infiltrates have also been reported [29–31]. Another CXL procedure in which the epithelium remains intact (also called “epithelium-on” or transepithelial CXL) was subsequently introduced [32]. This procedure has been shown to reduce patients’ pain, accelerate visual recovery, and avoid the potential risks of epithelial removal. Verified results including visual acuity and K values have been reported with transepithelial CXL [8, 22, 33–41]; however, the clinical efficiency of this modified technique compared with the conventional procedure is controversial. To the best of our knowledge, no adequate comparison between the two CXL modalities has been published.

In our analysis, we considered, as the main outcome, changes in the maximum K value as well as the corneal K, representing the main markers for the definition of keratoconus progression [4, 25, 42]. With regard to our meta-analysis, K-avg decreased more after conventional CXL compared with transepithelial CXL. The flattening effect on topographic indices may due to the lower riboflavin absorption in the corneal stroma, with transepithelial than with conventional CXL. As shown by Wollensak *et al*. [43], with the epithelium intact, only one fifth of the biomechanical effect may be achieved, compared with the conventional CXL. Experimental studies have also shown that the corneal epithelium may not only limit UVA transmittance, but also restrict riboflavin distribution in the stroma.

Keratometry and elevation readings (including anterior and posterior ones) are two separate indices to assess the progression of keratoconus; hence, it can be concluded that the measurement of elevation is an important way to demonstrate the long-term effects of CXL and its beneficial effect on corneal shape [44]. However, we didn’t find any study comparing posterior central elevation or posterior mean elevation between conventional and transepithelial CXL.

The posterior surface measurement can objectively evaluate the morphological features of the cornea. Previous studies have confirmed that changes in the posterior corneal surface elevation can be used to diagnose early keratoconus [45]. In patients with progressive keratoconus, such changes can be used as an indicator of the degree of the disease. Kranitz K *et al*. reported that posterior elevation proved to be the most sensitive parameter for detecting corneal shape changes after CXL [46]. Increases in posterior elevation could be considered as a progression signal of keratoconus. In some cases, a front surface flattening could be observed, at the same time as progressive back cornea surface elevation [47, 48]. In such circumstances, stabilization of the anterior part of the cornea can be achieved, but a potential ongoing ectatic change in the deeper layers of cornea should be taken into account [47, 48]. Further research is needed to confirm the changes in this parameter.

We found that the methodology of the transepithelial CXL protocol varied among the included studies. Mastropasqua *et al*. [18] performed the transepithelial treatment only as the standard treatment without epithelium debridement, with a UVA exposure of 370 nm for 30 minutes, at an irradiance of 3 mW/cm^2^. Soeters *et al*. also conducted a similar procedure [21]. Nawaz *et al*. used an improved protocol involving the administration of eye drops containing proparacaine to loosen the epithelium [19]. Bikbova *et al*. performed riboflavin soaking using an iontophoresis device to induce absorption [17]. An iontophoresis device was also applied by Lombardo *et al*., while the UVA irradiation was enhanced to shorten the exposure time [16]. However, the total energy density was equal among studies. Studies conducted by Mastropasqua *et al*. [18], Nawaz *et al*. [19], Rossi *et al*. [20], Soeters *et al*. [21] and Bikbova *et al*. [17] used a 3-mW/cm2 irradiation device for 30 minutes, while the study conducted by Lombardo *et al*. [15, 16] used 10-mW/cm2 irradiation for 9 minutes. Therefore, the total irradiation dose was approximately 5.4 J/cm2, in each included study. In the study of Schumacher *et al*., the effect of cross-linking was related to the total energy, and rapid UV cross-linking treatment was regarded as equivalent to the standard procedure [49]. Therefore, we believe that although the procedure parameters used were different, the intensity of the cross-linking effect on the cornea is similar. In this study, the RCTs included used the same irradiation energy and were compared according to whether the corneal epithelium was preserved or not.

The conventional CXL treatment is not recommended without performing a safety evaluation. We demonstrated that a significant difference in the CCT occurred after the conventional CXL procedure, compared with the transepithelial one. The outcome for the CCT value varied among studies; for studies with decreased CCT [17, 20, 21], the corneal-thinning effect was milder in the transepithelial CXL-treated group, while for studies with increased CCT [15, 19], the corneal-thickening effect was more expanded. A significant reduction of pachymetry in the early stage postoperatively and a statistically significant increase in later stages have been previously established [50, 51]. Postoperative keratocyte apoptosis and structural changes in corneal collagen amino-terminal side chains, as well as proteoglycans of the extracellular matrix may play a pivotal role in corneal thinning post-CXL treatment [46, 52]. According to Wollensak *et al*. [3], a cytotoxic keratocyte damage can occur after conventional CXL, throughout all layers of the stroma, influencing a depth of 320 to 350 μm, and even the endothelium, and thus inevitably harming corneal thickness. The decrease in corneal pachymetry, either at the apex or at the thinnest point, might indicate an ongoing change due to progressive keratoconus (especially when combined with an increase in the frontal surface K and increased elevation of the anterior and posterior corneal surface). Nevertheless, none of the included studies presented the change of corneal pachymetry and epithelium thickness separately, rendering the attribution of this change to the remolded epithelium or to changes in pachymetry thickness difficult. By preserving the epithelium, the damage to the endothelium can be spared and the safety of treatment increased, thus providing a safer option for patients with a thin cornea.

Our study has several strengths. To the best of our knowledge, it is the first meta-analysis comparing the treatment efficacy of conventional and transepithelial CXL for patients with keratoconus. Our quantitative evaluation was based on prospective RCTs studies, thus minimizing potential confounding bias. The extraction of data, study quality assessment, and data analysis were performed by two investigators, while arbitrators checked the consistency of the data, which highlights the accuracy of data in the meta-analysis.

Our study has also several limitations. The main weakness of our meta-analysis was the low number of included studies. Only two studies compared K-cyl and IOP outcomes between the conventional and transepithelial CXL-treated groups; therefore, the results should be interpreted with caution. Moreover, there was some heterogeneity in some results; however, the sensitivity analysis determined the source of all heterogeneity among the results. Publication bias might also have influenced the results. Especially the results of ECD indicated potential publication bias; however, the "trim and fill" analysis showed that there was only one potentially missing study, and, despite that, the results remained unchanged. Therefore, an individual-level meta-analysis, including a larger number of well-designed RCTs is needed to verify our results.

## Conclusions

Although transepithelial CXL appeared to be less efficient at flattening the K value compared with traditional conventional CXL, safety was improved by the preservation of the epithelium, and the CCT was less affected. The results of this meta-analysis indicate that the transepithelial CXL technique still needs modification.

## Supporting information

S1 File Search strategy for each database. (DOC)

S1 Table Exclusion studies list and exclusion reason. (DOC)

S2 Table PRISMA checklist. (DOC)

## Abbreviations

CCT: central corneal thickness
CDVA: corrected distance visual acuity
CI: confidence interval
CXL: collagen cross-linking
ECD: endothelial cell density
K: keratometry
K-avg: average K value
K-cyl: cylinder K value
K-flattest: flattest K value
K-steepest: steepest K value
RCTs: randomized controlled trials
SE: spherical equivalent
UDVA: uncorrected distance visual acuity
WMD: weighted mean difference
