# Supplementary material for: Conventional and transepithelial corneal cross-linking for patients with keratoconus
Source: PLoS One. 2018 Apr 5;13(4):e0195105. doi: 10.1371/journal.pone.0195105 (PMC5886478; doi:10.1371/journal.pone.0195105)
Supplement: S1 File — (DOC) [file pone.0195105.s001.doc]

**Search strategy for each databse**

MEDLINE (pubmed) search strategy

((((((((("randomized controlled trial"[Publication Type] OR "randomized controlled trials as topic"[MeSH Terms] OR "randomized controlled trial"[All Fields] OR "randomised controlled trial"[All Fields]) OR ("random allocation"[MeSH Terms] OR ("random"[All Fields] AND "allocation"[All Fields]) OR "random allocation"[All Fields] OR "randomized"[All Fields])) OR randomised[All Fields]) OR ("random allocation"[MeSH Terms] OR ("random"[All Fields] AND "allocation"[All Fields]) OR "random allocation"[All Fields] OR "random"[All Fields])) OR randomly[All Fields]) OR groups[All Fields]) OR controlled[All Fields]) OR ("clinical trials as topic"[MeSH Terms] OR ("clinical"[All Fields] AND "trials"[All Fields] AND "topic"[All Fields]) OR "clinical trials as topic"[All Fields] OR "trial"[All Fields]) OR ("Trials"[Journal] OR "trials"[All Fields])) AND ((((((cross-linking[All Fields] OR crosslinking[All Fields]) OR cross-linkage[All Fields]) OR cross-linking[All Fields]) OR (cross[All Fields] AND linking[All Fields] AND agents[All Fields])) OR CXL[All Fields]) OR CCL[All Fields])) AND ((("keratoconus"[MeSH Terms] OR "keratoconus"[All Fields]) OR keratoectasia[All Fields]) OR ("dilatation, pathologic"[MeSH Terms] OR ("dilatation"[All Fields] AND "pathologic"[All Fields]) OR "pathologic dilatation"[All Fields] OR "ectasia"[All Fields]))

Appendix 2. EMBASE (OvidSP) search strategy

1. exp randomized controlled trial/

2. exp randomization/

3. random$.tw.

4. or/1-3

5. exp clinical trial/

6. (clin$ adj3 trial$).tw.

7. random$.tw.

8. exp experimental design/

9. exp crossover procedure/

10. exp control group/

11. or/5-10

12. exp comparative study/

13. exp prospective study/

14. (control$ or prospectiv$ or volunteer$).tw.

15. or/12-14

16. 4 or 11 or 15

17. exp keratoconus/

18. keratoconus.tw.

19. keratoectasia.tw.

20. ectasia.tw.

21. or/17-20

22. crosslink$.tw.

23. cross linking.tw.

24. crosslinking.tw.

25. cross-linkage.tw.

26. cross-linking.tw.

27. cross-linking reagents.tw.

28. CCL.tw.

29. CXL.tw.

30. or/22-29

31. 16 and 21 and 30

Appendix 3 CENTRAL search strategy

#1 MeSH descriptor Keratoconus

#2 keratoconus

#3 keratoectasia

#4 (#1 OR #2 OR #3)

#5 cross linking

#6 crosslinking

#7 cross-linkage

#8 cross-linking

#9 crosslink*

#10 (#5 OR #6 OR #7 OR #8 OR #9)

#11 (#4 AND #10)
