# Supplementary material for: Conventional and transepithelial corneal cross-linking for patients with keratoconus
Source: PLoS One. 2018 Apr 5;13(4):e0195105. doi: 10.1371/journal.pone.0195105 (PMC5886478; doi:10.1371/journal.pone.0195105)
Supplement: S1 Table — (DOC) [file pone.0195105.s002.doc]

**S1 Table. Exclusion studies list and exclusion reason**

| Author | Article title | Detail | Exclusion reason |
| --- | --- | --- | --- |
| Suphi Taneri | Evaluation of epithelial integrity with various transepithelial corneal cross-linking protocols for treatment of keratoconus | J Ophthalmol, 2014, 2014: 614380. | Lack of data on comparison |
| Mashhoor F. Al Fayez | Transepithelial Versus Epithelium-Off Corneal Collagen Cross-Linking for Progressive Keratoconus: A Prospective Randomized Controlled Trial | Cornea, 2015, 34 Suppl 10: S53-56. | Lack of data on comparison |
| Antonella Franch | Evaluation of Intrastromal Riboflavin Concentration in Human Corneas after Three Corneal Cross-Linking Imbibition Procedures: A Pilot Study | J Ophthalmol, 2015, 2015: 794256. | Lack of data on comparison |
| Erdem Yuksel | Transepithelial Versus Epithelium-off Corneal Cross-Linking for the Treatment of Progressive Keratoconus: A Randomized Controlled Trial | Am. J. Ophthalmol., 2015, 160(2): 399-400. | Lack of data on comparison |
| David Touboul | Corneal confocal microscopy following conventional, transepithelial, and accelerated corneal collagen cross-linking procedures for keratoconus | J Refract Surg, 2012, 28(11): 769-76. | Lack of data on comparison |
| Carina Koppen | Refractive and topographic results of benzalkonium chloride–assisted transepithelial crosslinking | J Cataract Refract Surg, 2012, 38(6): 1000-5. | Other design (cohort) |
| Kocak, I. | Comparison of transepithelial corneal collagen crosslinking with epithelium-off crosslinking in progressive keratoconus | J Fr Ophtalmol, 2014, 37(5): 371-6. | Other design (retrospective) |
| Leopoldo Spadea | Recovery of corneal sensitivity after collagen crosslinking with and without epithelial debridement in eyes with keratoconus | J Cataract Refract Surg, 2015, 41(3): 527-32. | Other design (case series) |
| Paolo Vinciguerra | Transepithelial Iontophoresis Versus Standard Corneal Collagen Cross-linking: 1-Year Results of a Prospective Clinical Study | J Refract Surg, 2016, 32(10): 672-678. | Other design (non-randomized) |
| Eren C¸ erman | Transepithelial versus epithelium-off crosslinking in adults with progressive keratoconus | J Cataract Refract Surg, 2015, 41(7): 1416-25. | Other design (retrospective cohort) |
| Christine Wittig-Silva | A randomized controlled trial of corneal collagen cross-linking in progressive keratoconus: preliminary results | J Refract Surg, 2008, 24(7): S720-5. | Other design (no transepithelial CXL group) |
| Uri Elbaz | Accelerated versus standard corneal collagen crosslinking combined with same day phototherapeutic keratectomy and single intrastromal ring segment implantation for keratoconus | Br J Ophthalmol, 2015, 99(2): 155-9. | Other design (patient receive t-PTK or ICRS before CXL) |
| Sri Ganesh | Femtosecond Intrastromal Lenticular Implantation Combined With Accelerated Collagen Cross-Linking for the Treatment of Keratoconus--Initial Clinical Result in 6 Eyes | Cornea, 2015, 34(10): 1331-9. | Other design (case series) |
| Léa Jouve | Conventional and Iontophoresis Corneal Cross-Linking for Keratoconus: Efficacy and Assessment by Optical Coherence Tomography and Confocal Microscopy | Cornea, 2017;36:153-162 | Other design (non-randomized) |
| Rush Sloan W | Epithelium-off versus transepithelial corneal collagen crosslinking for progressive corneal ectasia: a randomised and controlled trial | Br J Ophthalmol, 2017, 101(4): 503-508. | Other design (corneal extasia) |
| Anastasios John Kanellopoulos | Hyperopic correction: clinical validation with epithelium-on and epithelium-off protocols, using variable fluence and topographically customized collagen corneal crosslinking | Clin Ophthalmol, 2014, 8: 2425-33. | Other design (hyperopic) |
| Nacim Bouheraous | Optical coherence tomography and confocal microscopy following three different protocols of corneal collagen-crosslinking in keratoconus | Invest. Ophthalmol. Vis. Sci., 2014, 55(11): 7601-9. | Other design (non-randomized) |
| George D. Kymionis | Long-term results of combined transepithelial phototherapeutic keratectomy and corneal collagen crosslinking for keratoconus: Cretan protocol | J Cataract Refract Surg, 2014, 40(9): 1439-45. | Other design (combine tPTK) |
| Aleksandar Stojanovic | Corneal collagen cross-linking with and without epithelial removal: a contralateral study with 0.5% hypotonic riboflavin solution | Biomed Res Int, 2014, 2014: 619398. | Other design (non-randomized) |
| George D. Kymionis | Combined transepithelial phototherapeutic keratectomy and corneal collagen crosslinking for ectatic disorders: cretan protocol | J Cataract Refract Surg, 2013, 39(12): 1939. | Other design (combine tPTK) |
| Stefano Baiocchi | Corneal crosslinking: riboflavin concentration in corneal stroma exposed with and without epithelium | J Cataract Refract Surg, 2009, 35(5): 893-9. |  |
| Kamil Bilgihan | Conventional Corneal Collagen Cross-Linking Versus Transepithelial Diluted Alcohol and Iontophoresis-Assisted Corneal Cross-Linking in Progressive Keratoconus | Cornea, 2017, 36(12): 1492-1497. | Other design (retrospectiv) |
| Xiangjun Chen | Epithelial Thickness Profile Change After Combined Topography-Guided Transepithelial Photorefractive Keratectomy and Corneal Cross-linking in Treatment of Keratoconus | J Refract Surg, 2016, 32(9): 626-34. | Other design (combine tPRK) |
| Hun Lee | Changes in posterior corneal elevations after combined transepithelial photorefractive keratectomy and accelerated corneal collagen cross-linking: retrospective, comparative observational case series | BMC Ophthalmol, 2016, 16: 139. | Other design (combine tPRK) |
| Alina Cantemir | Iontophoretic collagen cross-linking versus epithelium-off collagen cross-linking for early stage of progressive keratoconus - 3 years follow-up study | Acta Ophthalmol, 2017, 95(7): e649-e655. | Other design (retrospective) |
| Jyh Haur Woo | Woo Jyh Haur,Iyer Jayant Venkatramani,Lim Li et al. Conventional Versus Accelerated Collagen Cross-Linking for Keratoconus: A Comparison of Visual, Refractive, Topographic and Biomechanical Outcomes | Open Ophthalmol J, 2017, 11: 262-272. | Other design (retrospective) |
| Moonjung Choi | Comparison of the Conventional Dresden Protocol and Accelerated Protocol With Higher Ultraviolet Intensity in Corneal Collagen Cross-Linking for Keratoconus | Cornea, 2017, 36(5): 523-529. | Other design (both epithelium-off) |
| Mohammad Mehdi Sadoughi | Accelerated versus conventional corneal collagen cross-linking in patients with keratoconus: an intrapatient comparative study | Int Ophthalmol, 2016. | Other design (both epithelium-off) |
| Hun Lee | Changes in posterior corneal elevations after combined transepithelial photorefractive keratectomy and accelerated corneal collagen cross-linking: retrospective, comparative observational case series | BMC Ophthalmol, 2016, 16: 139. | Other design (combine tPRK) |
| Alex L. K. Ng, MRCSEd | Comparison of the Central and Peripheral Corneal Stromal Demarcation Line Depth in Conventional Versus Accelerated Collagen Cross-Linking.[J] . | Cornea, 2015, 34(11): 1432-6. | Other design (both epithelium-off) |
| Jeannette Beckman Rehnman | Treatment Effect and Corneal Light Scattering With 2 Corneal Cross-linking Protocols: A Randomized Clinical Trial | JAMA Ophthalmol, 2015, 133(11): 1254-60. | Other design (treat with contact lens) |
| Sara Brittingham | Corneal cross-linking in keratoconus using the standard and rapid treatment protocol: differences in demarcation line and 12-month outcomes | Invest. Ophthalmol. Vis. Sci., 2014, 55(12): 8371-6. | Other design (both epithelium-off) |
| Ronald R. Krueger | First proposed efficacy study of high versus standard irradiance and fractionated riboflavin/ultraviolet a cross-linking with equivalent energy exposure | Eye Contact Lens, 2014, 40(6): 353-7. | Other design (both epithelium-off) |
| Refik Oltulu | Intraoperative corneal thickness monitoring during corneal collagen cross-linking with isotonic riboflavin solution with and without dextran | Cornea, 2014, 33(11): 1164-7. | Other design (both epithelium-off) |
| [Young-Mi Chung](https://synapse.koreamed.org/ORCID/0000-0001-8365-8825) | Comparative study of two collagen membranes for guided tissue regeneration therapy in periodontal intrabony defects: a randomized clinical trial | J Periodontal Implant Sci, 2014, 44(4): 194-200. | Other design (both epithelium-off) |
| Uri Elbaz | Accelerated versus standard corneal collagen crosslinking combined with same day phototherapeutic keratectomy and single intrastromal ring segment implantation for keratoconus | Br J Ophthalmol, 2015, 99(2): 155-9. | Other design (both epithelium-off) |
| Banu Torun Acar | Can the effect of transepithelial corneal collagen cross-linking be improved by increasing the duration of topical riboflavin application? An in vivo confocal microscopy study | Eye Contact Lens, 2014, 40(4): 207-12. | Other design (non-randomized) |
| Minoru Tomita | Accelerated versus conventional corneal collagen crosslinking | J Cataract Refract Surg, 2014, 40(6): 1013-20. | Other design (comparative study) |
| Hassan Hashemi | Comparison of clinical results of two pharmaceutical products of riboflavin in corneal collagen cross-linking for keratoconus | Daru, 2014, 22: 37. | Other design (parallel clinical trial) |
| George D. Kymionis | Evaluation of corneal stromal demarcation line depth following standard and a modified-accelerated collagen cross-linking protocol | Am. J. Ophthalmol., 2014, 158(4): 671-675.e1. | Other design (both epithelium-off) |
| Adriano Magli | Epithelium-off corneal collagen cross-linking versus transepithelial cross-linking for pediatric keratoconus | Cornea, 2013, 32(5): 597-601. | Pediatric keratoconus |
| Muhsin Eraslan | Efficacy of Epithelium-Off and Epithelium-On Corneal Collagen Cross-Linking in Pediatric Keratoconus | Eye Contact Lens, 2017, 43(3): 155-161. | Pediatric keratoconus |
